# Postural awareness and its relation to pain: validation of an innovative instrument measuring awareness of body posture in patients with chronic pain

**DOI:** 10.1186/s12891-018-2031-9

**Published:** 2018-04-06

**Authors:** Holger Cramer, Wolf E. Mehling, Felix J. Saha, Gustav Dobos, Romy Lauche

**Affiliations:** 1 Department of Internal and Integrative Medicine, Kliniken Essen-Mitte, Faculty of Medicine, University of Duisburg-Essen, Essen, Germany; 2 Australian Research Centre in Complementary and Integrative Medicine (ARCCIM), University of Technology Sydney, Sydney, Australia; 3 Department of Family and Community Medicine, Osher Center for Integrative Medicine, University of California San Francisco, San Francisco, California, USA

**Keywords:** Awareness, Chronic pain, Posture

## Abstract

**Background:**

Habitual postural patterns are associated with musculoskeletal pain, and improving a maladaptive posture requires postural awareness in order to lead to clinical improvements. This study aimed to develop and evaluate the psychometric properties of an innovative postural awareness scale.

**Methods:**

A 12-item Postural Awareness Scale (PAS) was developed and administered to 512 chronic pain patients (50.3 ± 11.4 years, 91.6% female, 37.1% spinal/shoulder pain) to assess its factor structure and reliability. To determine convergent validity, measures of body awareness, body responsiveness, body image, and mindfulness were correlated with the PAS, as were clinical measures of pain intensity, disability, and mental health. Sensitivity to change was assessed in 202 outpatients participating in a 10-week multimodal mind-body program.

**Results:**

Factor analysis revealed two factors (*Ease/Familiarity with Postural Awareness* and *Need for Attention Regulation with Postural Awareness)* that explained 50.8% of the variance. Cronbach’s alpha for the complete scale was 0.80; Spearman-Brown coefficient of split-half reliability was 0.67; and intra-class correlation was ICC_2,1_ = 0.75 (95% confidence interval = 0.71, 0.78). Significant positive correlations were found for body awareness (*r* = 0.23), body responsiveness (*r* = 0.41), body image (*r* = 0.22–0.32), and mindfulness (*r* = 0.38); negative correlations for pain intensity (*r* = − 0.14), disability (*r* = − 0.12), depression (*r* = − 0.23), and stress (*r* = − 0.29). Postural awareness scores increased with a mind-body program (*p* < 0.001); changes in the PAS were negatively correlated with changes in pain intensity (*r* = − 0.35) in patients with spinal/shoulder pain.

**Conclusion:**

Self-reported postural awareness is associated with clinical symptoms in chronic pain patients; improvements in postural awareness are longitudinally associated with reduced pain in patients with spinal/shoulder pain.

## Background

Body posture refers to the position of a person’s body in space, the alignment of body parts in relationship to one another and to the environment at one point in time, and is influenced by each of the body’s joints [1, 2]. Postural control refers to building up posture against gravity and to ensuring that balance is maintained. It enables postural stabilization during voluntary movements and recovery of balance after disturbance [3]. Postural control also constructs a reference frame for proprioception, i.e. the perception of joint angles and muscle tensions, of movement, balance and posture [4]. The awareness of body posture relates to the awareness of proprioception, i.e. the subjective, phenomenological aspect of proprioception that enters conscious awareness [5]. Postural awareness, as we define it here, is the subjective conscious awareness of body posture that is mainly based on proprioceptive feedback from the body periphery to the central nervous system.

A large body of interventions aims to improve posture and postural control in patients with chronic musculoskeletal pain [6–12] based on the long-held notion that ‘bad’ posture and ‘poor’ postural control are major contributors to pain conditions, particularly to back and neck pain [13]. Previous research has indeed shown associations between spinal pain and posture, especially around chronic low back pain. Studies found that patients with chronic low back pain show altered muscle activation patterns of trunk muscles [7, 14–16], leading to decreased trunk movement [13], trunk stiffening [13], and postural instability [17], with those changes being overall indicative of dysfunctional postural control strategies [13, 18]. Studies also showed decreased proprioceptive acuity, i.e. patients with low back and neck pain have less accurate and precise positions sense [19–22], which indicates lower postural awareness in these patients. On the other hand, epidemiological studies not always support the notion that musculoskeletal pain is associated with body posture [23–25].

While the links between posture and pain are highly complex and controversially discussed, studies have shown that improving habitual postural patterns might lead to improvements in musculoskeletal pain conditions and prevent chronification or further deterioration [26–28]. Changing habitual patterns however requires postural awareness; this implies that postural awareness may be a key prerequisite in the process of adapting and maintaining healthy habitual postural and movement patterns in everyday life. Although numerous objective instrument-based measures of posture exist [29, 30], these can hardly be utilized outside of a laboratory setting and mostly measure posture under artificial conditions. To assess variations in everyday postural awareness without technical devices, self-report measures may be feasible, predictive of clinical changes and helpful as additional clinical and research tools. As, to our knowledge, no questionnaire currently exists to measure subjective postural awareness, this study aimed to develop and evaluate the psychometric properties of an innovative postural awareness scale for potential use in research and clinic. A second aim was to assess associations of pain intensity and pain-related variables with postural awareness. We hypothesized negative associations between the two categories of variables. Given the controversies regarding the influence of posture on chronic pain, we expected these associations to be only weak to moderate. Finally, the study aimed to assess associations of postural awareness with theoretically related instruments for measures of body awareness, body image and mindfulness. We hypothesized weak to moderate positive associations between these variables and postural awareness.

## Methods

### Sample and setting

This is a secondary analysis of data from two observational studies that were conducted at an academic medical center in Germany [31] (*Lauche, manuscript in preparation*). The primary aims of the observational studies was to investigate the effects of either a 2-week inpatient or a 10-week semi-stationary integrative medicine treatment on chronic pain. All patients with ICD-10 diagnoses of chronic pain conditions (chronic back and neck pain, fibromyalgia, headache, osteoarthritis, arthritis, and others), who were either admitted to hospital-based inpatient treatment or referred to an outpatient day care program at the same department between January 2013 and July 2014, were invited to participate. Written informed consent was obtained, and patients completed questionnaires at intake. Patients in the outpatient day care program completed additional questionnaires at discharge. The studies were approved by the University’s Ethics Committee and registered at a clinical trials register were the complete protocol for the studies can be found (clinicaltrials.gov; NCT01805947; NCT02038244) before patient recruitment.

### Measures

#### Postural awareness scale (PAS)

The PAS was developed in order to measure self-reported awareness of body posture in patients with chronic pain and potential changes in postural awareness induced by a multimodal mind-body training program. The original item pool was derived from qualitative interviews of patients with chronic non-specific neck pain undergoing a yoga program. Patients described detailed moment-to-moment variations in awareness of their body postures, perceived deficiencies in body awareness and their association with pain symptoms, as well as improvements in aspects of postural awareness after undergoing the yoga intervention [32]. Based on these interviews, 42 items were developed by the two of the authors (HC, RL) to survey postural awareness and perceived control of body posture. After a consensus process by two of the authors (HC, RL), 29 redundant items and items only loosely related to postural awareness were removed; and 13 items were included in the two above-described studies to field-test the preliminary PAS items. Item responses are scored on a 7-point Likert scale ranging from 1 (not at all true about me) to 7 (very true about me). Six items are reversely scored, so that higher score values consistently indicate higher postural awareness. The summary score ranges from 13 to 91.

For the assessment of convergent validity, the following scales were used:

#### Body awareness questionnaire (BAQ)

We used the German version of the BAQ, which measures attentiveness to normal, non-emotive internal bodily processes and sensations, specifically sensitivity to bodily cycles and rhythms, small changes in normal functioning, and anticipation of bodily reactions on 17 items scored on a 7-point Likert scale [33, 34] (*translated with permission by the author)*. This measure is one of the most commonly used and best-validated instruments for self-reported body awareness and has shown excellent reliability.

#### Body responsiveness scale (BRS)

The German version of the BRS is a 6-item instrument measuring responsiveness to bodily sensations on two scales for *Trust in Bodily Sensations* and *Perceived Connection between Mental and Physical Processes* using 7-point Likert scales [34, 35] (*translated with permission by the author)*.

#### Dresden body image inventory (Dresdner Körperbildfragebogen; DKB)

The DKB measures body image on five scales for *vitality*, *self-acceptance*, *sexuality*, *self-esteem*, and *physical contact* using 35 items scored on a 5-point Likert scale [36].

#### Conscious Presence and Self Control (CPSC)

A modified short-form of the Freiburg Mindfulness Inventory, the CPSC, measures mindfulness or situational awareness using 10 items on a 4-point Likert scale [37].

#### Pain visual analog scale (VAS)

Mean pain intensity during the past 4 weeks was measured on a 100-mm VAS ranging from 0 (no pain at all) to 100 (worst pain imaginable) [38]. This scale is international standard for self-reported pain intensity.

#### Pain perception scale (PPS)

The PPS measures subjectively felt pain on two scales: affective pain and sensory pain using 24 items on a 4-point Likert scale [39].

#### Pain disability index (PDI)

The PDI assesses functional disability, how pain interferes with specific aspects of a person’s life using 7 items on an 11-point Likert scale [40].

#### Beck depression inventory (BDI)

The BDI assesses depression by 21 items using 4-point Likert scales [41, 42] and is worldwide one of the most commonly used depression scales.

#### Perceived stress scale (PSS)

Self-perceived stress levels in specific situations during the last month were assessed on the 10-item German version of the PSS using a 4-point Likert scale [43].

### Statistical analysis

#### Construct validity

Exploratory factor analysis using principle components extraction was performed to explore the factor structure of the PAS. Since most items were intercorrelated (*r* = − 0.107 to 0.667), an Oblimin rotation was used. Factors were extracted if their eigenvalue was > 1. Domain scores of any resulting factors and of a total score were calculated as a sum of the component item scores.

#### Reliability

Cronbach’s α, alpha if item deleted, item-scale correlations, and item difficulty were calculated for each factor and the total score for internal consistency of the PAS. Split-half reliability was assessed as the Spearman-Brown coefficient; and two-way random intra-class correlation (ICC_2,1_) with 95% confidence intervals [44] were used to assess agreement between measures.

#### Convergent validity

In order to assess convergent validity, the strength of relationship of the PAS with theoretically related instruments for measures of body awareness, body image and mindfulness was assessed. Pearson’s correlation coefficients between the instrument and the BAQ, BRS, DKB and CPSC were calculated.

#### Postural awareness in patients with chronic pain

To assess postural awareness in patients with chronic pain, the instrument’s total score and/or subscale scores were assessed and differences between samples (spinal/shoulder pain versus other pain diagnoses), settings (inpatients versus outpatients) and genders (men versus women) were tested using independent *t*-tests. Associations of the PAS with clinical measures of pain (VAS, PPS, and PDI), mood (BDI) and perceived stress (PSS) were assessed by Spearman’s correlation coefficients. Additionally, independent predictors of postural awareness were assessed by linear forward stepwise multiple regression analyses with linear outcome and linear or dichotomous predictors. Only variables that were significantly correlated with the summary score of the respective instrument or subscale in univariate analysis were included in the initial regression model.

#### Responsiveness

The instrument’s sensitivity to change with a mind-body intervention was assessed in the outpatient day care program sample. After baseline assessment, these patients participated in a 10-week multimodal mind-body intervention [45]. The 60-h program, consisting of stress management training, moderate exercise, Mediterranean diet, and cognitive behavioral techniques with a focus on self-care strategies, was delivered for 6 h once a week over 10 weeks. The intervention was based on both the mind–body program of the Benson–Henry Mind/Body Medical Institute, Harvard Medical School [46] and the Mindfulness-Based Stress Reduction (MBSR) program of the University of Massachusetts [47, 48]. A number of activities expected to influence postural awareness, such as mindfulness training, yoga and qigong, were incorporated [45]. Scores of the PAS before and after this mind-body program were compared using paired-sample *t*-tests; and correlated to changes in pain intensity using Pearson’s correlation coefficient.

All statistics were performed using the statistical package IBM SPSS Statistics (Version 22.0; IBM Inc., New York, USA). A *p*-value of < 0.05 (two-tailed) was considered significant in all analyses.

## Results

### Sociodemographic characteristics

A total of 512 patients (91.6% female; mean age 50.3 ± 11.4 years) were included in the analyses. Across both studies, patients’ average pain intensity was 45.2 ± 26.1 mm on the VAS; mean pain duration was 12.9 ± 11.9 years (Table 1).

**Table 1:** Sociodemographic and clinical characteristics of the study sample (mean ± standard deviation)

	Total (*N* = 512)	Inpatient sample (*n* = 310)	Outpatient sample (*n* = 202)
Sociodemographic characteristics			
Age, in years	50.3 ± 11.4	50.7 ± 12.4	49.6 ± 9.7
Gender			
Female, *n* (%)	469 (91.6%)	282 (91.0%)	187 (92.6%)
BMI, in kg/m^2^	26.4 ± 5.6	26.9 ± 5.7	25.7 ± 5.2
Family status, *n* (%)			
Single	87 (17.0%)	57 (18.4%)	30 (14.9%)
With partner/married	338 (66.0%)	193 (62.3%)	145 (71.8%)
Divorced, separated, widowed	81 (15.8%)	54 (17.4%)	27 (13.4%)
Education, *n* (%)			
< High school	279 (54.5%)	171 (55.2%)	108 (53.5%)
At least high school	124 (24.2%)	73 (23.5%)	51 (25.2%)
University	106 (20.7%)	63 (20.3%)	43 (21.3%)
Employment, *n* (%)			
Full-time	170 (33.2%)	95 (30.6%)	75 (37.1%)
Part-time	132 (25.8%)	72 (23.22%)	60 (29.7%)
Unemployed	26 (5.1%)	20 (6.5%)	6 (3.0%)
Home keeper	32 (6.3%)	21 (6.8%)	11 (5.4%)
Retired	96 (18.8%)	68 (21.9%)	28 (13.9%)
Sick leave	48 (9.4%)	27 (8.7%)	21 (10.4%)
In education	3 (0.6%)	2 (0.6%)	1 (0.5%)
Applied for disability pension, *N* (%)	83 (16.2%)	52 (16.8%)	31 (15.3%)
Clinical characteristics			
Pain condition, *n* (%)^a^			
Headache			
Migraine	105 (20.5%)	39 (12.6%)	66 (32.7%)
Tension type headache	33 (6.4%)	11 (3.5%)	22 (10.9%)
Other headache	80 (15.6%)	50 (16.1%)	30 (14.9%)
Rheumatic diseases			
Fibromyalgia	114 (22.3%)	71 (22.9%)	43 (21.3%)
Osteoarthritis	90 (17.6%)	57 (18.4%)	33 (16.3%)
Rheumatoid arthritis	17 (3.3%)	10 (3.2%)	7 (3.5%)
Spinal/shoulder pain			
Low back pain	150 (29.3%)	82 (26.5%)	68 (33.7%)
Neck pain	48 (9.4%)	29 (9.4%)	19 (9.4%)
Shoulder pain	55 (10.7%)	31 (10.0%)	24 (11.9%)
Other pain	265 (51.8%)	175 (56.5%)	90 (44.6%)
Duration of pain, in years	12.9 ± 11.9	11.0 ± 10.7	15.6 ± 13.0
Intensity of pain, 0-100 mm VAS	45.2 ± 26.1	48.2 ± 25.2	40.7 ± 26.9

^a^More than one pain condition per patient possible

### Descriptive scale characteristics, factor structure and reliability

Mean item values for the PAS ranged from 2.5 to 5.5 (Table 2). With a Kaiser-Meyer-Olkin’s measure of sampling adequacy of 0.82, the sample was suitable for factor analysis. Primary component factor analysis revealed a three-factor structure explaining 55.6% of the variance (Table 2). However, only one item (PAS 3) exclusively loaded on factor 3; this item was thus removed from the field test analyses, leaving two factors explaining 50.8% of the variance. The complete scale had internal consistency Cronbach’s α of 0.80, a Spearman-Brown coefficient of 0.67, and intra-class correlation of ICC_2,1_ = 0.75, 95% confidence interval [44] = 0.71 to 0.78. For factor 1, which describes high, yet effortless postural awareness and connectedness (*Ease/Familiarity with Postural Awareness*), Cronbach’s α was 0.81, Spearman-Brown coefficient was 0.71, and intra-class correlation was ICC_2,1_ = 0.80, 95% CI = 0.78 to 0.83. For factor 2, which describes low levels of postural awareness and connectedness that require high efforts (*Need for Attention Regulation with Postural Awareness*), Cronbach’s α was 0.77, Spearman-Brown coefficient was 0.80, and intra-class correlation was ICC_2,1_ = 0.81, 95% CI = 0.78 to 0.83 (Table 2).

**Table 2:** Descriptive scale characteristics, factor structure, and reliability of the Postural Awareness Scale (PAS)

				Factor 1: *Ease/Familiarity with Postural Awareness* (alpha = 0.81)			Factor 2: *Need for Attention Regulation with Postural Awareness* (alpha = 0.77)		
	Item	Mean ± standard deviation	Item difficulty	Factor loading	Alpha if item deleted	Corrected item-total correlation	Factor loading	Alpha if item deleted	Corrected item-total correlation
1	Needs to concentrate for being aware of posture^a^	3.6 ± 1.7	0.51	–	–	–	0.80	0.72	0.58
2	Awareness of bad posture only by pain^a^	2.9 ± 1.7	0.41	–	–	–	0.78	0.72	0.59
3	Knows how to change posture to relieve pain^b^	5.0 ± 1.5	0.71	–	–	–	–	–	–
4	Slumps down when sitting^a^	2.8 ± 1.7	0.40	–	–	–	0.65	0.73	0.54
5	Unaware of posture when focused^a^	2.5 ± 1.3	0.36	–	–	–	0.54	0.75	0.48
6	Difficulties to consciously adopt a posture^a^	3.9 ± 1.9	0.56	–	–	–	0.72	0.73	0.54
7	Often checks posture when working	3.7 ± 1.7	0.53	0.73	0.78	0.57	–	–	–
8	Influences her/his own appeal by posture	4.9 ± 1.6	0.70	0.56	0.81	0.40	–	–	–
9	Always aware of sitting or standing posture	3.4 ± 1.5	0.49	0.80	0.76	0.67	–	–	–
10	Often makes her/himself aware of her/his posture	3.8 ± 1.6	0.54	0.79	0.75	0.67	–	–	–
11	Aware of posture even when focused	2.8 ± 1.5	0.40	0.77	0.77	0.62	–	–	–
12	Regulates how she/he feels through posture	3.6 ± 1.6	0.51	0.63	0.80	0.49	–	–	–
13	Needs to concentrate to feel whether a posture benefits her/him or not^a^	3.4 ± 1.6	0.49	–	–	–	0.51	0.77	0.38

^a^Reversed scoring

^b^Item excluded from final instrument

### Convergent validity

The two subscales of the PAS were weakly correlated (*r* = 0.29, *p* < 0.001). In line with our hypotheses, the PAS total score as well as the two subscales were weakly to moderately correlated with body responsiveness (both subscales of the BRS), body image (all subscales of the DKB) and mindfulness (CPSS). The total score and *Ease/Familiarity with Postural Awareness* were additionally correlated with body awareness (Table 3).

**Table 3:** Pearson’s correlations of the Postural Awareness Scale (PAS) with measures of body awareness, body responsiveness, body image, and mindfulness. [* indicates *p* < 0.05]

	Body Awareness Questionnaire	Body Responsiveness Scale		Dresden Body Image Inventory					Conscious Presence and Self Control
		Trust in Bodily Sensations	Perceived Connection between Mental and Physical Processes	Vitality	Self-accep-tance	Sexuality	Self-esteem	Physical contact	
Total sample									
PAS total	0.23^*^	0.41^*^	0.26^*^	0.26^*^	0.32^*^	0.22^*^	0.25^*^	0.24^*^	0.38^*^
*Ease/Familiarity with Postural Awareness*	0.37^*^	0.48^*^	0.09^*^	0.23^*^	0.28^*^	0.16^*^	0.23^*^	0.11^*^	0.36^*^
*Need for Attention Regulation with Postural Awareness*	0.01	0.17^*^	0.32^*^	0.19^*^	0.24^*^	0.19^*^	0.18^*^	0.11^*^	0.25^*^
Spinal/shoulder pain									
PAS total	0.29^*^	0.46^*^	0.12	0.34^*^	0.30^*^	0.20^*^	0.20^*^	0.17^*^	0.41^*^
*Ease/Familiarity with Postural Awareness*	0.46^*^	0.48^*^	−0.00	0.32^*^	0.25^*^	0.15^*^	0.18^*^	0.10	0.42^*^
*Need for Attention Regulation with Postural Awareness*	0.02	0.29^*^	0.20^*^	0.24^*^	0.25^*^	0.19^*^	0.14	0.18^*^	0.28^*^

When limiting the sample to patients with spinal or shoulder pain, the results of the correlational analyses were comparable to those of the total sample (Table 3).

### Postural awareness in patients with chronic pain

The mean PAS total score [range 12–84] was 41.2 ± 10.9, the *Ease/Familiarity with Postural Awareness* subscale [range 6–42] 22.2 ± 6.8 and the *Need for Attention Regulation with Postural Awareness* subscale [range 6–42] 19.1 ± 6.8. The total score and both subscale scores did not differ between setting, diagnosis, or gender (Table 4). In line with our hypotheses, the PAS total score as well as *Ease/Familiarity with Postural Awareness* were weakly correlated with lower pain intensity, pain disability, depression, and stress; and *Need for Attention Regulation with Postural Awareness* with lower pain intensity, depression, and stress (Table 5). In linear regression analyses, the PAS total score.

**Table 4:** Total score of the Postural Awareness Scale (PAS) (mean ± standard deviation) in the complete sample and differences between inpatients and outpatients; between different pain diagnoses; and between men and women

	Total sample (*n* = 512)	Inpatient sample (*n* = 310)	Outpatient sample (*n* = 202)	P	Spinal/ shoulder pain (*n* = 190)	Other pain (*n* = 322)	*P*	Men (*n* = 42)	Women (*n* = 470)	*P*
PAS total	41.2 ± 10.9	41.5 ± 10.5	40.9 ± 11.6	0.54	40.8 ± 11.2	41.5 ± 10.8	0.541	42.5 ± 10.6	41.1 ± 11.0	0.45
*Ease/Familiarity with Postural Awareness*	22.2 ± 6.8	22.1 ± 6.7	22.3 ± 6.9	0.76	22.0 ± 6.8	22.2 ± 6.8	0.754	22.9 ± 6.5	22.1 ± 6.8	0.46
*Need for Attention Regulation with Postural*	19.1 ± 6.8	19.4 ± 6.7	18.6 ± 7.0	0.20	19.2 ± 6.9	18.8 ± 6.7	0.505	19.6 ± 6.3	19.0 ± 6.9	0.63

**Table 5:** Pearson’s correlations of the Postural Awareness Scale (PAS) with clinical measures of pain and mood for the total sample and for the subgroup of patients with spinal or shoulder pain. Asterisks indicate significant correlations

	Pain Intensity	Pain Perception Scale		Pain Duration	Pain Disability Index	Beck Depression Inventory	Perceived Stress Scale
		Affective pain	Sensory pain				
Total sample							
PAS total	−0.14^*^	−0.08	−0.02	0.06	−0.12^*^	−0.23^*^	−0.29^*^
*Ease/Familiarity with Postural Awareness*	−0.14^*^	− 0.05	0.01	0.06	−0.13^*^	− 0.14^*^	− 0.24^*^
*Need for Attention Regulation with Postural Awareness*	− 0.09^*^	− 0.08	− 0.05	0.04	− 0.07	−0.23^*^	− 0.23^*^
Spinal/shoulder pain							
PAS total	−0.16^*^	−0.04	− 0.02	0.04	− 0.21^*^	−0.12	− 0.23^*^
*Ease/Familiarity with Postural Awareness*	−0.19^*^	− 0.07	0.06	− 0.03	−0.22^*^	− 0.07	−0.19^*^
*Need for Attention Regulation with Postural Awareness*	−0.08	−0.00	0.01	0.01	−0.13	−0.13	− 0.20^*^

(*R*^*2*^ *= 0.31*) was independently associated with lower pain intensity; and higher body awareness, body responsiveness, mindfulness, and vitality (Table 6). The *Ease/Familiarity with Postural Awareness* Subscale (*R*^*2*^ *= 0.31*) was independently associated with lower pain intensity; and higher body awareness, Trust in bodily sensations, mindfulness, and vitality (Table 6). The *Need for Attention Regulation with Postural Awareness* Subscale (*R*^*2*^ *= 0.18*) was independently associated with lower depression; and higher body responsiveness, and mindfulness (Table 6).

**Table 6:** Linear regression analyses: independent predictors of postural awareness

Dependent variable	Predictor variable	B ± SE	β	*P*
PAS Total				
	Pain intensity	−0.79 ± 0.02	−0.14	< 0.001
	BAQ	0.07 ± 0.03	0.11	0.017
	BRS Factor 1	0.64 ± 0.11	0.27	< 0.001
	BRS Factor 2	0.67 ± 0.15	0.19	< 0.001
	CPSC	0.11 ± 0.03	0.18	< 0.001
	DKB Vitality	1.90 ± 0.73	0.11	< 0.001
	Constant	14.34 ± 3.00	–	< 0.001
Ease/Familiarity with Postural Awareness				
	Pain intensity	−0.49 ± 0.01	−0.15	< 0.001
	BAQ	0.07 ± 0.03	0.18	0.017
	BRS Factor 1	0.46 ± 0.07	0.16	< 0.001
	CPSC	0.45 ± 0.07	0.32	< 0.001
	DKB Vitality	1.26 ± 0.43	0.17	0.004
	Constant	5.64 ± 1.78	–	0.002
Need for Attention Regulation with Postural Awareness				
	BDI	−0.10 ± 0.04	−0.12	0.016
	BRS Factor 1	0.19 ± 0.07	0.13	0.005
	BRS Factor 2	0.59 ± 0.09	0.28	< 0.001
	CPSC	0.04 ± 0.02	0.11	0.026
	Constant	36.85 ± 1.68	–	0.002

When limiting the sample to patients with spinal or shoulder pain, the results of the correlational analyses were more or less comparable; however correlations with pain disability were stronger and those with depression no longer were significant (Table 4). In this sample, the PAS total score (*R*^*2*^ *= 0.33*) was independently associated with trust in bodily sensations, mindfulness, and vitality. The *Ease/Familiarity with Postural Awareness* Subscale (*R*^*2*^ *= 0.29*) was independently associated with lower pain intensity and perceived stress; and higher body awareness (*p* = 0.034), trust in bodily sensations, and self-esteem. The *Need for Attention Regulation with Postural Awareness* Subscale (*R*^*2*^ *= 0.22*) was independently associated with lower perceived stress, and higher trust in bodily sensations.

### Responsiveness

After participation at the mind-body program, the PAS total score as well as both subscales significantly increased (Table 7). Likewise, pain intensity decreased after participation (Table 7); and changes in pain intensity were negatively associated with changes in the PAS total score (*r* = − 0.17; *p* = 0.026). However, this correlation was driven by patients with spinal or shoulder pain (*r* = − 0.35, *p* = 0.004) and not significant for patients with other pain diagnoses (*r* = − 0.06, *p* = 0.58).

**Table 7:** Sensitivity to change: values of the Postural Awareness Scale (PAS) and pain intensity (visual analog scale) (mean ± standard deviation) before and after participation in the mind-body group program. (*p* values for within-group pre-post changes by t-tests for dependent samples)

	Week 0 (*n* = 202)	Week 10 (*n* = 168)	*P*
PAS total	40.9 ± 11.6	46.0 ± 11.4	< 0.001
*Ease/Familiarity with Postural Awareness*	22.3 ± 6.9	25.7 ± 6.9	< 0.001
*Need for Attention Regulation with Postural Awareness*	18.6 ± 7.0	20.3 ± 7.1	0.003
Pain intensity	54.5 ± 19.9	35.5 ± 20.5	< 0.001

## Discussion

### Summary

The analysis revealed a two-factor structure of the postural awareness scale (PAS) after exclusion of one item in two samples of chronic pain patients. The two scales were comprised of six items each, describing high yet effortless postural awareness and connectedness (Factor 1, *Ease/Familiarity with Postural Awareness*) and postural awareness and connectedness that require high efforts (Factor 2, *Need for Attention Regulation with Postural Awareness*). The questionnaire has shown overall acceptable psychometric properties, with good internal consistency for the overall instrument, and at least acceptable consistency for the two subscales. Hypotheses-conform correlations were found for pain intensity as well as for measures of mindfulness, body awareness and body image. The PAS showed further sensitivity to change in chronic pain patients undergoing a multimodal outpatient treatment program, and those changes were correlated to changes in pain intensity in the chronic back, neck, and shoulder pain patients. However, these findings should be regarded only as preliminary, and the PAS clearly needs further validation. In order to finally judge the PAS’ diagnostic value, dimensionality (e.g. using Rasch analysis) and a confirmatory factor analysis using the proposed 2-factor solution are necessary.

### Factor structure

The instrument has a two-factor structure, *Ease/Familiarity with Postural Awareness* and *Need for Attention Regulation with Postural Awareness.* Based on the included items, the two factors might be interpreted as two opposite ends of a continuum of effort necessary to becoming aware of one’s posture. However, the two factors were not only clearly differentiated in factor analyses, but they also showed clearly different patterns of relationship to other variables, potentially indicating different mechanisms of action.

### Relationship to other measures

The factor *Ease/Familiarity with Postural Awareness* showed significant correlations with the *Body Awareness Question*naire, the subscale *Trust in Bodily Sensations* of the *Body Responsiveness Scale,* and the *Conscious Presence and Self Control questionnaire*. While the BAQ and the CPSC measure the degree of attentiveness towards bodily processes, the BRS also examines the responsiveness to bodily sensations rather than perception alone (i.e. connectedness). The *Ease/Familiarity with Postural Awareness subscale* reflect high postural awareness, i.e. high sensitivity to bodily cues, and connectedness to the body. Hence, the common denominator between the scales may be a trait of perceptual sensitivity and connectedness to the body more generally; independent of whether it is of interoceptive, exteroceptive, or proprioceptive nature.

The second factor *Need for Attention Regulation with Postural Awareness* however is only slightly correlated to the BAQ and the BRS subscale *Trust in Bodily Sensations*, but instead to the BRS subscale *Perceived Connection between Mental and Physical Processes*. *Perceived Connection between Mental and Physical Processes* reflects a common direction of an individual’s plans or wishes and their bodily desires, i.e. a low level of conflict between their cognitions and emotions on the one hand and their bodily needs on the other hand (*Cramer* et al.*, in preparation*). High levels of *Perceived Connection between Mental and Physical Processes*, just as *Need for Attention Regulation with Postural Awareness*, would thus reflect a detachment between mind and body, which requires an effort to balance conscious cognitive processes and bodily needs. In the subsample of spinal/shoulder pain, the relationship to the BRS subscale *Trust in Bodily Sensations* is also stronger indicating that the instrument may have higher validity in patients with spinal pain alone.

Interestingly, *both factors* were also correlated to subscales of the Dresden Body Image Inventory, which measures the body image, i.e. positive or negative attitudes towards the body and appearance. This correlation could reflect an association between high levels of awareness of one’s body and a generally more positive attitude towards it. Individuals experiencing themselves as disconnected from their own body can be expected to have a more negative or ambivalent attitude toward their bodily features and appearance as has been demonstrated e.g. for patients with eating disorders [49].

No major differences in PAS scores have been found between male and female patients, in- and outpatients, and pain conditions (spinal/shoulder pain vs. other pain). It can only be assumed that patients with chronic pain conditions might share some common features regarding body awareness and connectedness. While postural awareness has mainly been investigated in low back pain, it is reasonable that postural dysfunction might be related to a variety of musculoskeletal conditions, including osteoarthritis (with or without knee replacement) [50, 51] or fibromyalgia [52, 53]. However, changes in postural awareness seem to be related to improved symptoms in patients with spinal or shoulder pain only: the PAS has been shown sensitive to change, i.e. after an intervention targeting body awareness (including postural awareness) significant increases in the total score as well as in both subscales have been observed. This means that participants reported increased postural awareness, and that they needed less effort to perceive their bodily posture. Nevertheless, changes were negatively correlated to changes in pain intensity in the subsample of patients with spinal or shoulder pain only, i.e. increased postural awareness may be an influencing factor for pain amelioration in this patient population but not in other chronic pain patients.

This study has several limitations: First, the studies were observational trials not primarily conducted for validation purposes. Second, the sample consisted of participants with a variety of chronic pain conditions. Third, the sample further consisted of over 90% female participants. Fourth, a number of possible additional steps in questionnaire development (e.g. external expert review, language level considerations, and pilot testing on a patient sample including assessment of comprehensibility, time burden and completeness of content) were not performed. Fifth, the longitudinal data were uncontrolled, which precludes any causal inferences. Finally, no objective measure of postural sensitivity/control was included as a potential gold standard, against which the construct validity of the new scale could be assessed. We therefore cannot make a definitive statement about an association between self-reported postural awareness and actual postural control.

## Conclusion

Despite these limitations, the Postural Awareness Scale (PAS) was field-tested in a large sample of pain patients, including a variety of measures for cross-validation. The PAS represents the first instrument to measure subjective postural awareness with acceptable psychometric properties. Postural awareness is associated with pain intensity, physical and mental impairments in patients with chronic pain; and improvements in postural awareness through multimodal interventions were associated with improvements in pain intensity. However, further validation in an independent sample and against objective measures is imperative before the PAS can be used in routine clinical practice and research with chronic pain patients, preferably in addition to objective measures.

## Abbreviations

BAQ: Body awareness questionnaire
BDI: Beck depression inventory
BRS: Body responsiveness scale
CPSC: Conscious presence and self control
DKB: Dresdner körperbildfragebogen
IBM: International business machines corporation
ICC: intra-class correlation
ICD: International statistical classification of diseases and related health problems
PAS: Postural awareness scale
PDI: Pain disability index
PPS: Pain perception scale
PSS: Perceived stress scale
SPSS: Statistical package for the social sciences
VAS: Visual analog scale
